# A founder *COL4A4* pathogenic variant resulting in autosomal recessive Alport syndrome accounts for most genetic kidney failure in Romani people

**DOI:** 10.3389/fmed.2023.1096869

**Published:** 2023-02-08

**Authors:** Pavlina Plevova, Jana Indrakova, Judy Savige, Petra Kuhnova, Petra Tvrda, Dita Cerna, Sarka Hilscherova, Monika Kudrejova, Daniela Polendova, Radka Jaklova, Martina Langova, Helena Jahnova, Jana Lastuvkova, Jiri Dusek, Josef Gut, Marketa Vlckova, Pavla Solarova, Gabriela Kreckova, Eva Kantorova, Jana Soukalova, Rastislav Slavkovsky, Jana Zapletalova, Tomas Tichy, Dana Thomasova

**Affiliations:** ^1^Department of Clinical and Molecular Pathology and Medical Genetics, University Hospital Ostrava, Ostrava, Czechia; ^2^Department of Biomedical Sciences, Faculty of Medicine, University of Ostrava, Ostrava, Czechia; ^3^Department of Medicine (Melbourne Health and Northern Health), The University of Melbourne, Royal Melbourne Hospital, Melbourne, Australia; ^4^Department of Medical Genetics, Faculty of Medicine in Plzeň, Charles University and University Hospital Plzeň, Plzeň, Czechia; ^5^Department of Medical Genetics, Thomayer University Hospital, Prague, Czechia; ^6^Department of Pediatrics, Third Faculty of Medicine, Charles University and University Hospital Královské Vinohrady, Prague, Czechia; ^7^Department of Medical Genetics, Krajská zdravotní, a.s., Masaryk Hospital in Ústí nad Labem, Ústí nad Labem, Czechia; ^8^Department of Pediatrics, 2nd Faculty of Medicine, Charles University and Motol University Hospital, Prague, Czechia; ^9^Department of Pediatrics, Hospital Česká Lípa, Česká Lípa, Czechia; ^10^Department of Biology and Medical Genetics, 2nd Faculty of Medicine, Charles University and Motol University Hospital, Prague, Czechia; ^11^Department of Medical Genetics, University Hospital Hradec Králové, Hradec Králové, Czechia; ^12^Department of Medical Genetics, Gennet, s.r.o., Liberec, Czechia; ^13^Department of Medical Genetics, Hospital České Budějovice a.s., České Budějovice, Czechia; ^14^Department of Medical Genetics and Genomics, University Hospital Brno, Brno, Czechia; ^15^Institute of Molecular and Translational Medicine, Faculty of Medicine and Dentistry, Palacký University Olomouc, Olomouc, Czechia; ^16^Department of Medical Biophysics, Faculty of Medicine and Dentistry, Palacký University Olomouc, Olomouc, Czechia; ^17^Institute of Clinical and Molecular Pathology, Faculty of Medicine and Dentistry, Palacký University Olomouc, Olomouc, Czechia

**Keywords:** Alport syndrome, Romani, hematuria, proteinuria, end-stage kidney failure, hearing loss, consanguinity

## Abstract

**Introduction:**

Romani people have a high prevalence of kidney failure. This study examined a Romani cohort for pathogenic variants in the *COL4A3, COL4A4*, and *COL4A5* genes that are affected in Alport syndrome (AS), a common cause of genetic kidney disease, characterized by hematuria, proteinuria, end-stage kidney failure, hearing loss, and eye anomalies.

**Materials and methods:**

The study included 57 Romani from different families with clinical features that suggested AS who underwent next-generation sequencing (NGS) of the *COL4A3, COL4A4, and COL4A5* genes, and 83 family members.

**Results:**

In total, 27 Romani (19%) had autosomal recessive AS caused by a homozygous pathogenic c.1598G>A, p.Gly533Asp variant in *COL4A4* (*n* = 20) or a homozygous c.415G>C, p.Gly139Arg variant in *COL4A3* (*n* = 7). For p.Gly533Asp, 12 (80%) had macroscopic hematuria, 12 (63%) developed end-stage kidney failure at a median age of 22 years, and 13 (67%) had hearing loss. For p.Gly139Arg, none had macroscopic hematuria (*p* = 0.023), three (50%) had end-stage kidney failure by a median age of 42 years (*p* = 0.653), and five (83%) had hearing loss (*p* = 0.367). The p.Gly533Asp variant was associated with a more severe phenotype than p.Gly139Arg, with an earlier age at end-stage kidney failure and more macroscopic hematuria. Microscopic hematuria was very common in heterozygotes with both p.Gly533Asp (91%) and p.Gly139Arg (92%).

**Conclusion:**

These two founder variants contribute to the high prevalence of kidney failure in Czech Romani. The estimated population frequency of autosomal recessive AS from these variants and consanguinity by descent is at least 1:11,000 in Czech Romani. This corresponds to a population frequency of autosomal dominant AS from these two variants alone of 1%. Romani with persistent hematuria should be offered genetic testing.

## Introduction

Alport syndrome (AS) is a genetic disease characterized by progressive kidney failure, sensorineural hearing loss, and ocular abnormalities (1). It results from pathogenic variants in the collagen IV genes that encode chains of the α3-4-5 heterotrimer (2). Pathogenic variants in the *COL4A5* gene result in X-linked AS, whereas biallelic *COL4A3* or *COL4A4* variants are found in autosomal recessive AS (AR AS) (3). Individuals with heterozygous *COL4A3* or *COL4A4* mutations have thin basement membrane nephropathy or autosomal dominant AS (AD AS) with typically normal kidney function, but proteinuria may occur in later life. The *COL4A3* and *COL4A4* heterozygotes represent carriers of AR AS (3–5).

Romani children are often seen with AS or end-stage renal disease (ESRD) in Slovakia (6, 7), a European country neighboring the Czech Republic. The origins of the Romani are unclear, but the current understanding is that they have come to Europe from the Indian subcontinent centuries ago (8, 9), and several studies describe a high incidence of ESRD in India and Indian immigrants in Europe (10–12). Founder variants in the Alport genes have been reported in other isolated populations such as Ashkenazi (13–15), and the p.Gly624Asp variant in *COL4A5* accounts for nearly half the variants found in eastern Europe (16, 17).

This study examined whether pathogenic variants in the Alport genes (*COL4A3, COL4A4, COL4A5*) were responsible for the high prevalence of ESRD in Romani people living in the Czech Republic.

## Materials and methods

This study included 57 self-identified Romani people from 57 families with clinical features that suggested AS and 83 family members who were known to be affected or at risk. They were recruited from the departments of pediatric nephrology, nephrology, or genetics from all regions of the Czech Republic between 1 January 2014 and 31 July 2022. This study was approved by the Institutional Ethics Committee of the University Hospital Ostrava according to the principles of the Declaration of Helsinki, and all study participants provided written informed consent.

Participants provided peripheral venous blood samples that were referred to the Laboratory of DNA diagnostics of the Department of Medical Genetics, University Hospital of Ostrava for genetic analysis. Genomic DNA was isolated using conventional techniques and underwent sequence capture-based, next-generation sequencing (NGS) of the *COL4A3, COL4A4*, and *COL4A5* genes. The sequencing included all the coding exons, at least 50 bp of flanking intronic sequence, and the untranslated regions of the *COL4A3* (NM_000091.5), *COL4A4* (NM_000092.5), and *COL4A5* (NM_033380.3) genes using NimbleGen SeqCap EZ Target Enrichment System (Roche, Switzerland) as described previously (18). Sequencing was performed on an Illumina platform (Illumina, CA, USA), and data were examined with the standard Illumina base-calling procedure. Mapping to the human genome sequence, variant-calling, and copy number variation (CNV) analyses were performed with FinalistDx (IAB, Czech Republic). Pathogenic variant validation was performed with direct Sanger sequencing using the ABI Big Dye Terminator Cycle Sequencing Detection Kit v.3.1 and an ABI 3130 genetic analyzer (Applied Biosystems, CA, USA) according to the manufacturer’s instructions. Multiplex Ligation-Dependent Probe Amplification (MLPA) was performed for the *COL4A3, COL4A4*, and *COL4A5* genes with SALSA kits P191/P192 (*COL4A5*), P439 (*COL4A3*), and P444 (*COL4A4*) (MRC-Holland, The Netherlands), and the results were analyzed using Coffalyzer software (MRC-Holland, The Netherlands).

DNA from three individuals was further examined for pathogenic variants by an NGS panel of 462 genes associated with genetic kidney diseases using the same strategy (Supplementary material 1). Targeted genotyping of parents and family members of affected individuals was performed where possible.

The genomic DNA of the first individual with a homozygous p.Gly533Asp variant in *COL4A4* was examined more in detail. This included a whole-genome microarray SNP analysis with HumanCytoSNP-12v2.1 BeadChips (Illumina, CA, USA) and examination of the array data with Bluefuse Multi Software (Illumina, CA, USA) according to the manufacturer’s instructions.

Clinical data for patients with confirmed AS were provided by the referring physicians from the medical records. Age at ESRD was defined as the age at the clinical diagnosis of kidney failure, and where this was not available, the age at commencing dialysis or at the first kidney transplant.

In addition, DNA from 300 non-Romani individuals with clinical features that suggested AS and their 225 family members were examined.

### Evaluation of the pathogenicity of sequence variants

The variants were described according to the recommendations of the Human Genome Variation Society (HGVS). Sequence variants were assessed according to the criteria of the American College of Medical Genetics (ACMG) (19). Pathogenicity was ascertained based on the following criteria: the presence of a missense variant involving a position 1 Gly or an in-frame Gly deletion in the collagen Gly-X-Y triple-helical domain; a splice-site or truncating variant; or a large genomic duplication or deletion (20). Variants were also assessed based on molecular, epidemiological, segregation, and computational criteria (Polyphen-2, SIFT, Mutation Taster, PhyloP100). The following online databases were searched to determine if variants were previously reported: Ensembl, gnomAD browser, the Human Gene Mutation Database (HGMD), Leiden Open (source) Variation Database (LOVD), and ClinVar. The potential impact of each variant was reported as one of the five categories: pathogenic, likely pathogenic, uncertain significance, likely benign, or benign.

### Statistical analysis

Qualitative parameters in groups with different genotypes were compared using Fisher’s exact test. Ages were compared using the Mann–Whitney *U* test. Statistical analysis was performed using SPSS version 23 software (Armonk, NY: IBM Corp., USA), and a *p*-value of less than 0.05 was considered significant.

## Results

The study cohort comprised 57 unrelated individuals, including 56 with both parents Romani and one with only one Romani parent. They included 23 (40%) men and 34 (60%) women, with a median age at the referral of 16 years (range, 4–57 years) for men and 15.5 years (range, 4–60 years) for women. In addition, 83 affected or at-risk family members were examined, including 81 patients where both parents were Romani. These included 42 (51%) men and 41 (49%) women with an overall median age of 29.0 years (range 2–63 years).

In the Romani patients, only two pathogenic variants were detected in the Alport genes. These were c.1598G > A in *COL4A4* corresponding to p.Gly533Asp and c.415G > C in *COL4A3* corresponding to p.Gly139Arg. No pathogenic or likely pathogenic variant was found in *COL4A5*. A girl with one Romani parent had the p.Gly533Asp variant in *COL4A4* together with another pathogenic variant in trans in *COL4A4* (Table 1).

**TABLE 1:** Summary of pathogenic sequence variants detected in Romani, one parent Romani, and non-Romani patients.

Genotype	Probands	Family genetic testing positive	All pts. with the variant (probands + family testing positive)	Family genetic testing negative
	**No. of pts.**	**No. of pts.**	**No. of pts. (% from the group)**	**No. of pts.**
**Romani**				
***COL4A4*** **c.1598G>A, p.Gly533Asp**				
Homozygous (=AR AS)	19	1	**20** (18)	23
Heterozygous (=AD AS)	24	44	**68** (62)	
Double heterozygous: + *COL4A3* p.Gly139Arg (=digenic AS)	1	0	**1** (1)	
***COL4A3*** **c.415G>C, p.Gly533Asp**				
Homozygous (=AR AS)	2	5	**7** (7)	1
Heterozygous (=AD AS)	4	9	**13** (12)	
All Romani	50	59	**109** (100)	24
**One parent Romani**				
***COL4A4*** **c.1598G>A, p.Gly533Asp**				
Compound heterozygous: + *COL4A4* c.3707G>A, p.Gly1236Glu (=AR AS)	1	0	**1** (33)	0
Heterozygous (=AD AS)	0	2	**2** (66)	0
All one parent Romani	1	2	**3** (100)	0
**Non-Romani**				
***COL4A4*** **c.1598G>A, p.Gly533Asp**				
Compound heterozygous: + *COL4A4* c.3514_3515delinsTGAAA, p.Gly1172Ter (=AR AS)	1	0	**1** (8)	0
Heterozygous (=AD AS)	4	8	**12** (92)	4
***COL4A3*** **c.415G>C, p.Gly533Asp**				
Homozygous, heterozygous (=AR AS, AD AS)	0	0	**0**	0
All non-Romani	5	8	**13** (100)	4
All pts from all subgroups	56	69	125	28

The above table shows the distribution of patients with homozygous and heterozygous p.Gly533Asp *COL4A4* and p.Gly139Arg *COL4A3* variants in the Romani (*n* = 109), one parent Romani (*n* = 3), and non-Romani (*n* = 13) patients. Homozygous (i.e., two identical) or compound heterozygous (i.e., two different) pathogenic variants in the same (*COL4A4* or *COL4A3*) gene cause AR AS; heterozygous (i.e., one) pathogenic variants in *COL4A4* or *COL4A3* gene cause AD AS; the occurrence of one (heterozygous) pathogenic variant in *COL4A4* and simultaneously one (heterozygous) pathogenic variant in *COL4A3* (i.e., double heterozygous status) causes digenic Alport syndrome; numbers of all patients with the particular genotype are in bold.

pts., patients; AR AS, autosomal recessive Alport syndrome; AD AS, autosomal dominant Alport syndrome; AS, Alport syndrome.

Both the p.Gly533Asp and p.Gly139Arg variants were considered to be pathogenic. These variants affected the position 1 glycine residue in the collagen Gly-X-Y triple-helical domain that was not adjacent to an interruption. They were not found in gnomAD. The computational tools all suggested pathogenicity (Polyphen-2– 0.999 and 1.000, respectively; SIFT–Damaging; Mutation Taster–Disease causing); the affected glycines were highly conserved with a PhyloP100 score of 4.763 and 5.3, respectively. The p.Gly533Asp was considered pathogenic in ClinVar, likely pathogenic or pathogenic in LOVD, and was reported as pathogenic for AR AS in two patients from the UK (21). The p.Gly139Arg had not been reported in ClinVar but was considered pathogenic in LOVD (22). Both variants segregated with the disease in the families.

**FIGURE 1:**
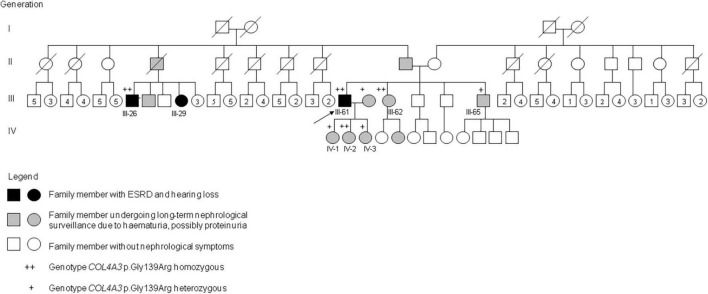
Pedigree of a Romani family with AR AS due to homozygous *COL4A3* c.415G>C, p.Gly139Arg. Genotypes and phenotypes of selected family members: III-26: *COL4A3* p.Gly139Arg homozygous; ESRD at 42 years, dialysis, hearing loss, died at 54 years. III-29: no genetic testing; long-term dialysis, age 52 years. III-61: *COL4A3* p.Gly139Arg homozygous; ESRD at 17 years, dialysis since 20 years, kidney transplantation at 33 and 41 years, hearing loss at 35 years, lens exchange for anterior lenticonus at 41 years; III-62: *COL4A3* p.Gly139Arg homozygous; microscopic hematuria and small proteinuria diagnosed at 10 years, moderate to severe hearing loss since 46 years, normal kidney function at 53 years. III-65: *COL4A3* p.Gly139Arg heterozygous; transient acute kidney failure at 41 years after long-term physical and psychical overload, 3 months later isolated microscopic hematuria. IV-1: *COL4A3* p.Gly139Arg heterozygous; isolated microscopic hematuria diagnosed at 31 years. IV-2: *COL4A3* p.Gly139Arg homozygous; isolated microscopic hematuria diagnosed at 20 years. IV-3: *COL4A3* p.Gly139Arg heterozygous; isolated microscopic hematuria diagnosed at 22 years.

Autosomal recessive AS was confirmed genetically in 22 of the 57 individuals in the cohort with clinical features of AS (39%). Another six individuals with AR AS were detected among the family members (Table 1). The family tree of one family is shown in Figure 1. A further nine patients identified from the 300 individuals from the non-Romani Czech cohort also had AR AS. In total, the 28 Romani represented 76% (28/37) of all patients with AR AS diagnosed in our laboratory.

In addition, there were 83 individuals heterozygous for one of these variants, with 70 (84%) heterozygous for p.Gly533Asp in *COL4A4* and 13 (16%) for p.Gly139Arg in *COL4A3* (Table 1), consistent with the diagnosis of AD AS or thin basement membrane nephropathy. One 10-year-old girl with isolated microscopic hematuria diagnosed at the age of three was a double heterozygote for the two variants. Altogether, monoallelic or biallelic pathogenic *COL4A4* or *COL4A3* variants were detected in 51 of 57 Romani with clinical features of AS (89%) and 61 of their 83 relatives (73%). This corresponded to an estimated prevalence of AR AS due to homozygous copies of these *COL4A4* and *COL4A3* variants in the Romani population in the Czech Republic of at least one in 11,000. These results also corresponded to a population frequency of these founder variants in at least one in 3,800 of the Czech Romani and probably higher (Table 2). These results also mean that AD AS due to these variants affects at least one in 3,800 Czech Romani.

**TABLE 2:** Estimated prevalence of *COL4A4* and *COL4A3* variants in the Romani population in the Czech Republic.

	Romani		Non-Romani	
**Genotype or type of AS**	**No. of pts.**	**Prevalence per 300,000^a^**	**No. of pts.**	**Prevalence^b^**
*COL4A4* p.Gly533Asp homozygous	20	1:15,000	0	0
*COL4A4* p.Gly533Asp heterozygous	68	1:4,400	11	1:950,000
*COL4A4* p.Gly533Asp + *COL4A3* p.Gly139Arg, double heterozygous	1	1:300,000	0	0
*COL4A3* p.Gly139Arg homozygous	7	1:42,800	0	0
*COL4A3* p.Gly139Arg heterozygous	13	1:23,000	0	0
Autosomal recessive AS^c^	27	1:11,000	n.a.	n.a.
Autosomal dominant AS (TBMN)^d^	81	1:3,800	n.a.	n.a.
Digenic *COL4A4* + *COL4A3*	1	1:300 000	0	0

^a^The population of 300,000 Romani people in the Czech Republic was used for calculation.

^b^The population of 10,500,000 non-Romani people in the Czech Republic was used for calculation.

^c^*COL4A4* p.Gly533Asp homozygous or *COL4A3* p.Gly139Arg homozygous.

^d^*COL4A4* p.Gly533Asp heterozygous or *COL4A3* p.Gly139Arg heterozygous.

AS, Alport syndrome; n.a., non-applicable; TBMN, thin basement membrane nephropathy.

In a group of 300 non-Romani Czech individuals and their 225 relatives, there was one individual who was a compound heterozygote for p.Gly533Asp in *COL4A4* and another pathogenic *COL4A4* variant in *trans*, 12 patients heterozygous for p.Gly533Asp *COL4A4*, and none with p.Gly139Arg in *COL4A3* (Table 1).

DNA from three Romani patients with AD AS whose phenotype did not correspond typically to the genotype was further examined for pathogenic variants by an NGS panel of 462 genes associated with genetic kidney diseases. The first patient was heterozygous for p.Gly533Asp in *COL4A4* and had isolated proteinuria from the age of 2 years, hearing loss from the age of 14 years, and had developed ESRD at the age of 18 years. His sister also had isolated proteinuria from adolescence and developed ESRD at the age of 61 years. The second patient also heterozygous for p.Gly533Asp in *COL4A4* developed ESRD at the age of 26 years. The third one was a 6-year-old boy heterozygous for p.Gly139Arg in *COL4A3* with microscopic hematuria, persistent proteinuria, and transient macroscopic hematuria. Other concurrent genetic etiologies for kidney disease were suspected in these individuals. However, testing with NGS for a kidney panel of 462 genes did not reveal any other pathogenic variants.

Clinical features associated with AR AS are summarized in Table 3. For p.Gly533Asp, 12 homozygotes (63%) developed ESRD at a median age of 22 years (range 12 to 55), and 13 (67%) had hearing loss, while all 20 (100%) had microscopic hematuria, 12 (80%) had macroscopic hematuria in childhood, and 17 (94%) had proteinuria with a median age of onset 5 years. For p.Gly139Arg, three homozygotes (50%) developed kidney failure by a median age of 42 years (range 17 to 56), five (83%) had hearing loss, six (100%) had microscopic hematuria, but none had macroscopic hematuria (*p* = 0.023), and five (83%) had proteinuria with a median age of onset 20.5 years. There was only one person with a recognized ocular abnormality (lenticonus) and no data on the others.

**TABLE 3:** Summary of clinical features in Alport syndrome patients with respect to the genotype.

	Clinical features	Microscopic hematuria	Macroscopic hematuria	Proteinuria	ESRD	Hearing loss
	**Subgroup of patients according to the genotype and ethnic group**	**No. of pts. (% in the subgroup)**	**No. of pts. (% in the subgroup)**	**No. of pts. (% in the subgroup)**	**No. of pts. (% in the subgroup)**	**No. of pts. (% in the subgroup)**
**AR AS**						
**1.**	***COL4A4* p.Gly533Asp homozygous, Romani**	*n* = 20				
1.1	Feature: Yes	20 (100)	12 (80)	17 (94)	12 (63)	13 (67)
1.2	Feature: No	0	3 (20)	1 (6)	7 (37)	6 (33)
1.3	Feature: Information not available	0	5	2	1	1
1.4	Median age at feature diagnosis (years) (range)^a^	4.0 (8 m-6)	5.0 (2–12)	5.0 (1–13)	22.0 (15–55)	14.5 (10–20)
**2.**	***COL4A4* p.Gly533Asp + other *COL4A4* variant (compound heterozygous)** **one parent Romani, non-Romani**	*n* = 2				
2.1	Feature: Yes	2 (100)	2 (100)^b^	1 (50)^c^	0	1 (50)
2.2	Feature: No	0	0	1 (50)^d^	2	1 (50)
2.3	Feature: Information not available	0	0	0	0	0
2.4	Median age at feature diagnosis (years) (range)^a^	2.75 (2.5–3)	2.75 (2.5–3)	3 (3)	n.a.	n.a.
**3.**	***COL4A3* p.Gly139Arg homozygous, Romani**	*n* = 7				
3.1	Feature: Yes	6 (100)	0 (0)	5 (83)	3 (50)	5 (83)
3.2	Feature: No	0	6 (100)	1 (17)	3 (50)^e^	1 (17)
3.3	Feature: Information not available	1	1	1	1	1
3.4	Median age at feature diagnosis (years) (range)^a^	8.0 (5–20)	-	20.5 (10–31)	42.0 (17–56)	35.0 (25–46)
	Statistics 3.1 vs. 1.1 (Fisher’s exact test)	n.a.	***p* = 0.023**	*p* = 0.446	*p* = 0.653	*p* = 0.367
	Statistics 3.4 vs. 1.4 (Mann-Whitney *U* test)	***p* = 0.010**	n.a.	***p* = 0.044**	*p* = 0.347	***p* = 0.001**
**Digenic AS**						
**4.**	***COL4A4* p.Gly533Asp, *COL4A3* p.Gly139Arg double heterozygous Romani**	*n* = 1				
4.1	Feature: Yes	1	0	1	0	0
4.2	Feature: No	0	1	0	1	1
4.3	Feature: Information not available	0	0	0	0	0
4.4	Median age at feature diagnosis (years) (range)^a^	3	n.a.	3	n.a.	n.a.
**AD AS**						
**5.**	***COL4A4* p.Gly533Asp heterozygous Romani, one parent Romani**	*n* = 70 (68 Romani, 2 one parent Romani)				
5.1	Feature: Yes	61 (91)	1 (1)^f^	5 (7)^g^	3 (4)^h^	6 (9)^i^
5.2	Feature: No	6 (9)^j^	66 (99)	62 (93)	66 (96)	61 (91)
5.3	Feature: Information not available	3	3	3	1	3
5.4	Median age at feature diagnosis (years) (range)^a^	7.0 (1–58)	n.a.	n.a.	26.0 (18–61)	12.0 (1–35)
	Statistics 5.1 vs. 1.1 (Fisher’s exact test)	*p* = 0.329	***p* < 0.0001**	***p* < 0.0001**	***p* < 0.0001**	***p* < 0.0001**
	Statistics 5.4 vs. 1.4 (Mann-Whitney *U* test)	***p* < 0.0001**	n.a.	n.a.	*p* = 0.515	*p* = 0.420
**6.**	***COL4A4* p.Gly533Asp heterozygous Non-Romani**	*n* = 12				
6.1	Feature: Yes	11 (92)	0	2 (17)	0	2 (17)
6.2	Feature: No	1 (8)^k^	12 (100)	10 (83)^l^	12 (100)	10 (83)
6.3	Feature: Information not available	0	0	0	0	0
6.4	Median age at feature diagnosis (years) (range)^a^	9.0 (4–30)	n.a.	26.5 (23–30)	n.a.	3 (3)
	Statistics 6.1 vs. 5.1 (Fisher’s exact test)	*p* = 1.000	*p* = 1.000	*p* = 0.287	*p* = 1.000	*p* = 0.600
	Statistics 6.4 vs. 5.4 (Mann-Whitney *U* test)	*p* = 0.528	n.a.	n.a.	n.a.	n.a.
**7.**	***COL4A3* p.Gly139Arg heterozygous Romani**	*n* = 13				
7.1	Feature: Yes	11 (92)	1 (8)^m^	2 (17)^n^	0 (1 acute transient renal failure)^o^	1 (8)^p^
7.2	Feature: No	1 (8)^q^	11 (92)	10 (83)	12 (100)	11 (92)
7.3	Feature: Information not available	1	1	1	1	1
7.4	Median age at feature diagnosis (years) (range)^a^	14.0 (5–41)	6 (6)	31.0 (5–57^r^)	n.a.	n.a.
	Statistics 7.1 vs. 5.1 (Fisher’s exact test)	*p* = 1.000	*p* = 0.282	*p* = 0.287	*p* = 1.000	*p* = 1.000
	Statistics 7.4 vs. 5.4 (Mann-Whitney *U* test)	***p* < 0.0001**	n.a.	n.a.	n.a.	n.a.
	Statistics 7.1 vs. 6.1 (Fisher’s exact test)	*p* = 1.000	*p* = 1.000	*p* = 1.000	*p* = 1.000	*p* = 1.000
	Statistics 7.4 vs. 6.4 (Mann-Whitney *U* test)	***p* < 0.0001**	n.a.	n.a.	n.a.	n.a.

The above table shows the occurrence of clinical features of Alport syndrome in the patients with respect to the different types of AS, including AR AS, digenic AS and AD AS, to the particular genetic variant and ethnic type of population–Romani, one parent Romani and non-Romani. The recorded features include microscopic and macroscopic hematuria, proteinuria, end-stage renal disease, and hearing loss. The age of onset of each of the features was documented whenever possible. AR AS patients were divided into three groups according to the genotype. The occurrence and age of onset of the features was statistically analyzed between the patients with homozygous p.Gly533Asp *COL4A4* variant (subgroup 1, *n* = 20) and the patients with homozygous p.Gly139Arg *COL4A3* variant (subgroup 3, *n* = 7) using Fisher’s exact test, and Mann-Whitney U test, respectively. AR AS due to compound heterozygous *COL4A4* variants (subgroup 2, *n* = 2) could not be included in the statistical analysis because of the low number of patients. Digenic AS (subgroup 4) was found in only one patient, thus statistical analysis was also not possible. Patients with AD AS were divided into three groups. The data from Romani patients with heterozygous p.Gly139Arg *COL4A3* variant (subgroup 7, *n* = 13) were compared with those of Romani plus one parent Romani patients with heterozygous *COL4A4* p.Gly533Asp variant (subgroup 5, *n* = 70) and then to those of non-Romani patients with heterozygous *COL4A4* p.Gly533Asp (subgroup 6, *n* = 12). Again, the occurrence of the features was statistically analyzed using Fisher’s exact test, and the Mann-Whitney *U* test was used to compare the ages of onset of the features between the groups. As regards results of statistical analyses, a *p*-value less than 0.05 was considered significant (bold).

^a^Information not available in all pts.

^b^Transient, isolated attack accompanying a respiratory infection.

^c^Gross proteinuria at 11 years, renal biopsy disclosed focal glomerulosclerosis.

^d^Age 14 years.

^e^3 pts without ESRD at 25, 33, and 54 years.

^f^Since 9 years, no proteinuria.

^g^4 of them were members of the same family, 2 of them isolated proteinuria and ESRD at 18 and 61 years; other concurrent genetic etiology is possible but was not disclosed; NGS kidney gene panel of 462 genes testing did not reveal any other pathogenic variant in the pts with ESRD at 18 and 26 years.

^h^The pts. with ESRD at 18 and 61 years are relatives (see g).

^i^The pt with isolated proteinuria and ESRD at 18 years (see g, h)–hearing loss since 14 years; other etiology of hearing loss is probable in two siblings with moderate hearing loss since 1 year and in the other two patients.

^j^4 pts without features of AS at 3, 7, 29 and 40 years, 2 pts isolated proteinuria with ESRD (see g).

^k^Without features of AS at 20 years.

^l^In 1 pt, transient PU in pregnancy at 29 years.

^m^Transient, isolated attack.

^n^One of the pts also had transient macroscopic hematuria (see m); NGS kidney gene panel of 462 genes testing did not reveal any other pathogenic variant in the pt.

^o^The patient developed transient acute kidney failure at 41 years after long-term physical and psychical overload; 3 months later, isolated microscopic hematuria was present in this patient; he has persistent isolated microscopic hematuria and normal kidney function 1 year after the acute kidney failure episode.

^p^Other etiology of hearing loss is probable

^q^Without features of AS at 6 years.

^r^The 57-year-old pt was treated for diabetes mellitus for 30 years.

ESRD, end-stage renal disease; pts, patients; AR AS, autosomal recessive Alport syndrome; AD AS, autosomal dominant Alport syndrome; years, years; m, months; n.a., non-applicable.

Thus the p.Gly533Asp variant in *COL4A4* was associated with a more severe phenotype than p.Gly139Arg in *COL4A3*, with an earlier onset of proteinuria (*p* = 0.044), earlier onset of ESRD (*p* = 0.347), and a greater risk of macroscopic hematuria (*p* = 0.023). However, there was also a large variability in the severity of the clinical features even within a family.

A total of six (9%) Romani patients heterozygous for c.1598G>A in *COL4A4* did not have microscopic hematuria. Four (6%) did not have either microscopic hematuria or proteinuria or any other manifestation of AS. Two Romani siblings heterozygous for p.Gly533Asp *COL4A4* variant had isolated proteinuria and progressed to renal failure at 18 and 61 years, respectively. No other pathogenic variant was detected in one of the siblings by testing a panel of 462 genes for inherited kidney diseases, as mentioned above. Two other members of this family heterozygous for p.Gly533Asp had microscopic hematuria and concomitant proteinuria. They had normal renal functions at ages 36 and 40 years. Thus four of five patients with heterozygous p.Gly533Asp and proteinuria belonged to the same family.

Renal biopsy reports were available from eight patients with AR AS. The histological and electron microscopy findings included thin basement membrane nephropathy with the fusion of podocytes, thickening and splitting of the glomerular basement membrane, focal segmental glomerulosclerosis, or mesangioproliferative glomerulonephritis. Immunofluorescence α3 and α5 chain staining was negative in three patients with homozygous *COL4A4* p.Gly533Asp variant (Table 4).

**TABLE 4:** Summary of renal biopsy histology and α3, α5 chain immunofluorescence staining in patients with autosomal recessive Alport syndrome with respect to the genotype.

Renal biopsy details							
**Genotype**	**Renal biopsy report available** **no. of pts. (%)**	**TBMN, fusion of podocytes no. of pts. (% of pts. with renal biopsy report)**	**Thickening and splitting of the GBM no. of pts. (% of pts. with renal biopsy report)**	**Focal segmental glomerular sclerosis** **no. of pts. (% of pts. with renal biopsy report)**	**Mesangio-proliferative glomerulonephritis no. of pts. (% of pts. with renal biopsy report)**	**α3 chain immuno-fluorescence staining** **no. of pts. (% of pts. with renal biopsy report)**	**α5 chain immuno-fluorescence staining** **no. of pts. (% of pts. with renal biopsy report)**
*COL4A4 c.1598G*>*A*, p.Gly533Asp homozygous	7/20 (35)	2/7 (28)	2/7 (28)	2/7 (28)	1/7 (14)	3/7^1a,b,c^ (43)	2/7^1a,b^ (29)
*COL4A4* c.1598G>A, p.Gly533Asp + c.3707G>A, p.Gly1236Glu	1/1 (100)	0/1	0/1	0/1	1^2^/1 (100)	0/1	0/1
*COL4A4* c.1598G>A, p.Gly533Asp + c.3514 _3515delinsTGAAA, p.Gly1172Ter	0/1	n.a.	n.a.	n.a.	n.a.	n.a.	n.a.
*COL4A3* c.415G>C, p.Gly139Arg homozygous	0/7	n.a.	n.a.	n.a.	n.a.	n.a.	n.a.

^1a^1^st^ sample: α3: apparent defects of positivity in glomeruli, some glomeruli completely negative, Bowman’s capsule and tubular basement membranes (BM) negative, α5: glomerular BM negative, Bowman’s capsule BM positive, tubular BM only focally positive. ^1b^2^nd^ sample: α3: low expression intensity or complete defects of positivity in capillary glomerular loops, Bowman’s capsule BM positive, α5: staining not performed. ^1c^3^rd^ sample: α3 negative, α5 negative. ^2^segmental glomerular sclerosis also detected. Pts, patients; TBMN, thin basement membrane nephropathy; GBM, glomerular basement membrane; BM, basement membrane.

In patients with homozygous p.Gly533Asp in *COL4A4*, 45 homozygous benign variants (Supplementary material 2) were also detected, with 16 in *COL4A3* and 29 in *COL4A4.* Both are neighboring genes located in a head-to-head orientation. In the first patient, in whom a homozygous c.1598G > A *COL4A4* variant was detected, an SNP microarray analysis was performed. Loss of heterozygosity of 250.57 Mb (equivalent to 9% of the genome) was demonstrated in 17 regions, including a 47,837,907-bp loss of heterozygosity at the 2q32.1–2q37.1 region (chr2: 183,690,254–231,528,160), where the *COL4A3* and *COL4A4* genes are located. This suggested consanguinity of the third degree. Despite this fact, the parents of the patient claimed to be fourth-degree relatives because the grandfather of the patient’s mother was the brother of her father’s father. Fourth-degree relatives usually display a loss of heterozygosity of 80–90 Mb (23), while here we observed 250.57 Mb. This corresponds approximately to the first-cousin relationship.

## Discussion

In many European countries, Romani people constitute a major ethnic minority (7). According to the European Romani Rights Center, the estimated number of Romani in Czechia is between 250,000 and 300,000 (24), which represents 2.8% of the 10.5 million inhabitants. However, we have found that Romani represent 76% of all individuals with AR AS diagnosed in our laboratory and that this is due to two founder pathogenic variants and a high rate of consanguinity by descent.

Our data are similar to those observed in Slovakia, where Kolvek et al. frequently treated Romani children with ESRD (7). Although the estimated Romani population in Slovakia is at most 7% (8), 11 of their 14 patients with AS (79%) were Romani (7). They, too, hypothesized that this resulted from a founder effect (25). In an earlier report, AS has been reported in 14 families from Eastern Slovakia where about one in four were Romani (6). It is likely that p.Gly533Asp was responsible for many of their patients, as well as those reported in the UK (21) and the Netherlands (22) where Romani is also found. This variant has already been reported ten times in LOVD (22), which suggests that it is relatively common in Europe.

Based on our data, the estimated prevalence of AR AS in the Czech Romani population is at least 1:11,000 due to these founder variants. Thus, the corresponding heterozygous variants and AD AS occur in about 1% of the Romani population according to the Hardy–Weinberg equation. This is equivalent to the population frequency of AD AS due to multiple different variants in other populations. The true prevalence is likely to be even greater since there may be other less common variants also. Thus, individuals with heterozygous variants may be undetected. However, they too have a risk of hypertension, proteinuria, and renal impairment, and, even if small, the risk of ESRD should be assessed and monitored (26). The demonstration of p.Gly533Asp in *COL4A4* in the non-Romani population suggests that it will be widespread in Romani and non-Romani throughout Europe and maybe also in North America and Australia, wherever there has been Romani immigration.

Only two pathogenic variants were detected in the Romani in our study. The c.1598G>A variant in *COL4A4* corresponding to p.Gly533Asp was nearly four times more common than the c.415G>C variant in *COL4A3* corresponding to p.Gly139Arg. Two individuals with AR AS due to homozygous variants have been found in the UK (21), and this variant was also found in Czech people not known to be Romani. Based on our data, the *COL4A4* variant described here is the commonest cause of AR AS at least in the Czech Republic. Another variant c.3114C>G, p.Ser969Ter in *COL4A4* has been reported frequently in British people causing not only AR AS but also AD AS in the heterozygous form (27).

The p.Gly139Arg in *COL4A3* has not been reported previously. There was only one individual with both founder variants, which suggests that these occurred in different subpopulations of the Romani people. Another explanation is that the p.Gly139Arg is uncommon. This variant was only found in Romani. In Romani, AR AS was caused only by homozygous *COL4A4* c.1598G>A, whereas in non-Romani, c.1598G>A was found solely in combination with another pathogenic variant. This suggests the mixing of the Romani and Czech populations.

The degree of consanguinity in the Romani population may be higher than suspected because their population has been a relatively small community in the Czech Republic (7). This is important for clinicians to remember when undertaking genetic counseling for these families, and genetic testing and preconception analysis should be offered to the spouse or partner of a Romani who has the p.Gly533Asp or p.Gly139Arg variant.

The published data suggest that missense variants in *COL4A3* and *COL4A4* genes have a less severe phenotype than loss-of-function, splice site variants, or large deletions. Individuals with two truncating variants have an earlier onset of kidney failure or hearing loss than those with only one truncating variant, who are in turn more likely to develop ESRD than those with no truncating variant (5, 21, 28, 29). However, this genotype-phenotype correlation is not always seen (30).

The Romani cohort represents the opportunity to study modifying factors that worsen disease severity in AD AS. Overall the median age for the *COL4A4* variant was similar to that reported previously. However, both intra- and interfamilial variability in age at kidney failure has already been described (5, 31, 32), and was also seen here, with the age at ESRD varying from 17 to 42 and with normal renal function in another affected individual.

There are several large studies and a meta-analysis of genotype–phenotype analyses in individuals with AD AS (30–33). The likelihood of isolated microscopic hematuria, macroscopic hematuria, ESRD, and hearing loss in this cohort is similar to previous reports, but proteinuria occurred less often (5, 31). Early onset ESRD has been reported in AD AS, but it is rare and may result from coincidental diseases. For instance, in a meta-analysis of 777 individuals with AD AS, there were four patients with ESRD before the age of 25 years (31).

Overall, the variant features that are associated with a higher penetrance of hematuria are known. Both the Gly substitutions reported here are highly destabilizing variants, namely, Arg and Asp, and are consistent with an increased risk of hematuria (33).

Other AR diseases also occur in Romani including neuropathy, myopathy, and hearing loss (9, 34–36). It has been suggested that newborn screening for these diseases should be performed in Romani because of the sometimes 5% carrier rates (9).

In conclusion, this study identified two founder pathogenic variants, p.Gly533Asp in *COL4A4* and p.Gly139Arg in *COL4A3* in the Romani population. These variants explain the high proportion of Romani people among the Czech cohort with features suggesting AS. The estimated population frequency of AR AS from these variants is at least 1:11,000 in the Czech Romani. This corresponds to a population frequency of AD AS from these two variants alone of 1%. Our data suggest that consanguinity by descent is common in the Romani. Romani with persistent hematuria should be offered genetic testing, and preconception genetic testing should be offered to the partners of Romani who have one of these founder variants.

## Data availability statement

The datasets presented in this study can be found in online repositories. The names of the repository/repositories and accession number(s) can be found in the article/Supplementary material.

## Ethics statement

The studies involving human participants were reviewed and approved by Institutional Ethics Committee of the University Hospital Ostrava. Written informed consent to participate in this study was provided by the participants’ legal guardian/next of kin. Written informed consent was obtained from the individual(s) for the publication of any potentially identifiable images or data included in this article.

## Author contributions

PP, JI, PK, PT, DC, SH, MK, DP, RJ, ML, HJ, JL, JD, JG, MV, PS, GK, EK, JS, RS, JZ, TT, and DT performed the material preparation, data collection, and analysis. JS helped to analysis and writing. PP wrote the first draft of the manuscript and was the principal investigator of IP/RVO-FNOs/2015. DT was the principal investigator of NV19-06-00443. All authors contributed to the study conception and design, commented on previous versions of the manuscript, read, and approved the final manuscript.

## References

[1] Jais J Knebelmann B Giatras I Marchi M Rizzoni G Renieri A X-linked Alport syndrome: natural history in 195 families and genotype- phenotype correlations in males. *J Am Soc Nephrol.* (2000) 11:649–57. 10.1681/ASN.V114649 10752524

[2] Hudson B Tryggvason K Sundaramoorthy M Neilson E. Alport’s syndrome, Goodpasture’s syndrome, and type IV collagen. *N Engl J Med.* (2003) 348:2543–56. 10.1056/NEJMra022296 12815141

[3] Kashtan C Ding J Garosi G Heidet L Massella L Nakanishi K Alport syndrome: a unified classification of genetic disorders of collagen IV alpha345: a position paper of the Alport Syndrome Classification Working Group. *Kidney Int.* (2018) 93:1045–51. 10.1016/j.kint.2017.12.018 29551517

[4] Papazachariou L Papagregoriou G Hadjipanagi D Demosthenous P Voskarides K Koutsofti C Frequent COL4 mutations in familial microhematuria accompanied by later-onset Alport nephropathy due to focal segmental glomerulosclerosis. *Clin Genet.* (2017) 92:517–27. 10.1111/cge.13077 28632965

[5] Savige J Huang M Dabrera M Shukla K Gibson J. Genotype-phenotype correlations for pathogenic COL4A3–COL4A5 variants in X-linked, autosomal recessive, and autosomal dominant Alport syndrome. *Front Med.* (2022) 9:865034. 10.3389/fmed.2022.865034 35602506 PMC9120524

[6] Kluka V Tischler V Vereb J. [Alportś syndrome in Eastern Slovakia.] (Slovak). *CS Pediat.* (1986) 41:569–73. 3502944

[7] Kolvek G Podracka L Rosenberger J Stewart R van Dijk J Reijneveld S. Kidney diseases in Roma and non-Roma children from eastern Slovakia: are Roma children more at risk?. *Int J Public Health.* (2014) 59:1023–6. 10.1007/s00038-014-0609-z 25270618

[8] Zeman C Depken D Senchina D. Roma health issues: a review of the literature and discussion. *Ethn Health.* (2003) 8:223–49. 10.1080/1355785032000136434 14577997

[9] Kalaydjieva L Gresham D Calafell F. Genetic studies of the Roma (gypsies): a review. *BMC Med Genet.* (2001) 2:5. 10.1186/1471-2350-2-5 11299048 PMC31389

[10] Sakhuja V Sud K. End-stage renal disease in India and Pakistan: burden of disease and management issues. *Kidney Int Suppl.* (2003) 83:S115–8. 10.1046/j.1523-1755.63.s83.24.x 12864888

[11] Agarwal S Srivastava R. Chronic kidney disease in India: challenges and solutions. *Nephron Clin Pract.* (2009) 111:c197–203. 10.1159/000199460 19194110

[12] Lightstone L. Preventing renal disease: the ethnic challenge in the United Kingdom. *Kidney Int Suppl.* (2003) 83:S135–8. 10.1046/j.1523-1755.63.s83.29.x 12864893

[13] Barker D Pruchno C Jiang X Atkin C Stone E Denison J A mutation causing Alport syndrome with tardive hearing loss is common in the western United States. *Am J Hum Genet.* (1996) 58:1157–65. 8651292 PMC1915056

[14] Barker D Denison J Atkin C Gregory M. Common ancestry of three Ashkenazi-American families with Alport syndrome and COL4A5 R1677Q. *Hum Genet.* (1997) 99:681–4. 10.1007/s004390050429 9150741

[15] Webb B Brandt T Liu L Jalas C Liao J Fedick A A founder mutation in COL4A3 causes autosomal recessive Alport syndrome in the Ashkenazi Jewish population. *Clin Genet.* (2014) 86:155–60. 10.1111/cge.12247 23927549

[16] Zurowska A Bielska O Daca-Roszak P Jankowski M Szczepańska M Roszkowska-Bjanid D Mild X-linked Alport syndrome due to the COL4A5 G624D variant originating in the Middle Ages is predominant in Central/East Europe and causes kidney failure in midlife. *Kidney Int.* (2021) 99:1451–8. 10.1016/j.kint.2020.10.040 33309955

[17] Gibson J Fieldhouse R Chan M Sadeghi-Alavijeh O Burnett L Izzi V Genomics England research consortiumprevalence estimates of predicted pathogenic *COL4A3-COL4A5* variants in a population sequencing database and their implications for Alport syndrome. *J Am Soc Nephrol.* (2021) 32:2273–90. 10.1681/ASN.2020071065 34400539 PMC8729840

[18] Stehlikova K Skalova D Zidkova J Mrazova L Vondravek P Mazanec R Autosomal recessive limb-girdle muscular dystrophies in the Czech Republic. *BMC Neurol.* (2014) 14:154. 10.1186/s12883-014-0154-7 25135358 PMC4145250

[19] Richards S Aziz N Bale S Bick D Das S Gastier-Foster J ACMG laboratory quality assurance committee. Standards and guidelines for the interpretation of sequence variants: a joint consensus recommendation of the American college of medical genetics and genomics and the association for molecular pathology. *Genet Med.* (2015) 17:405–24. 10.1038/gim.2015.30 25741868 PMC4544753

[20] Liu J Wei X Li A Cui Y Xia X Qin W Novel mutations in COL4A3, COL4A4, and COL4A5 in Chinese patients with Alport Syndrome. *PLoS One.* (2017) 12:e0177685. 10.1371/journal.pone.0177685 28542346 PMC5436713

[21] Storey H Savige J Sivakumar V Abbs S Flinter F. COL4A3/COL4A4 mutations and features in individuals with autosomal recessive Alport syndrome. *J Am Soc Nephrol.* (2013) 24:1945–54. 10.1681/ASN.2012100985 24052634 PMC3839543

[22] Fokkema I Taschner P Schaafsma G Celli J Laros J den Dunnen JT. LOVD v.2.0: the next generation in gene variant databases. *Hum Mutat.* (2011) 32:557–63. 10.1002/humu.21438 21520333

[23] Wiszniewska J Bi W Shaw C Stankiewicz P Kang S Pursley A Combined array CGH plus SNP genome analyses in a single assay for optimized clinical testing. *Eur J Hum Genet.* (2014) 22:79–87. 10.1038/ejhg.2013.77 23695279 PMC3865406

[24] Romové, v Èeské republice. *Czech radio [Romani in Czech Republic] (Czech) (1997–2023).* Available online at: http://romove.radio.cz/cz/clanek/18884 (Accessed August 31, 2023).

[25] Kolvek G Rosicova K Rosenberger J Podracka L Stewart R Nagyova I End-stage renal disease among Roma and non-Roma: ROMA are at risk. *Int J Public Health.* (2012) 57:751–4. 10.1007/s00038-012-0365-x 22552750

[26] Savige J Lipska-Zietkiewicz B Watson E Hertz J Deltas C Mari F Guidelines for genetic testing and management of Alport syndrome. *Clin J Am Soc Nephrol.* (2022) 17:143–54. 10.2215/CJN.04230321 34930753 PMC8763160

[27] Dagher H Wang Y Fassett R Savige J. Three novel COL4A4 mutations resulting in stop codons and their clinical effects in autosomal recessive Alport syndrome. *Hum Mutat.* (2002) 20:321–2. 10.1002/humu.9065 12325029

[28] Oka M Nozu K Kaito H Fu X Nakanishi K Hashimura Y Natural history of genetically proven autosomal recessive Alport syndrome. *Pediatr Nephrol.* (2014) 29:1535–44. 10.1007/s00467-014-2797-4 24633401

[29] Lee J Nozu K Choi D Kang H Ha I Cheong H. Features of autosomal recessive Alport syndrome: a systematic review. *J Clin Med.* (2019) 8:178. 10.3390/jcm8020178 30717457 PMC6406612

[30] Kamiyoshi N Nozu K Fu X Morisada N Nozu Y Ye M Genetic, clinical, and pathologic backgrounds of patients with autosomal dominant Alport syndrome. *Clin J Am Soc Nephrol.* (2016) 11:1441–9. 10.2215/CJN.01000116 27281700 PMC4974872

[31] Matthaiou A Poulli T Deltas C. Prevalence of clinical, pathological and molecular features of glomerular basement membrane nephropathy caused by COL4A3 or COL4A4 mutations: a systematic review. *Clin Kidney J.* (2020) 13:1025–36. 33391746 10.1093/ckj/sfz176 PMC7769542

[32] Furlano M Martinez V Pybus M Arce Y Crespi J Venegas M Clinical and genetic features of autosomal dominant Alport syndrome: a cohort study. *Am J Kidney Dis.* (2021) 78:560–70. 10.1053/j.ajkd.2021.02.326 33838161

[33] Gibson J Huang M Shenelli Croos Dabrera M Shukla K Rothe H Hilbert P Genotype-phenotype correlations for COL4A3-COL4A5 variants that result in Gly substitutions in Alport syndrome. *Sci Rep.* (2022) 12:2722. 10.1038/s41598-022-06525-9 35177655 PMC8854626

[34] Bouwer S Angelicheva D Chandler D Seeman P Tournev I Kalaydjieva L. Carrier rates of the ancestral Indian W24X mutation in GJB2 in the general gypsy population and individual subisolates. *Genet Test.* (2007) 11:455–8. 10.1089/gte.2007.0048 18294064

[35] Safka Brozkova D Lastuvkova J Stepankova H Krutova M Trkova M Myska P DFNB49 is an important cause of non-syndromic deafness in Czech Roma patients but not in the general Czech population. *Clin Genet.* (2012) 82:579–82. 10.1111/j.1399-0004.2011.01817.x 22097895

[36] Safka Brozkova D Varga L Uhrova Meszarosova A Slobodova Z Skopkova M Soltysova A Variant c.2158-2A>G in MANBA is an important and frequent cause of hereditary hearing loss and beta-mannosidosis among the Czech and Slovak Roma population- evidence for a new ethnic-specific variant. *Orphanet J Rare Dis.* (2020) 15:222. 10.1186/s13023-020-01508-3 32847582 PMC7448337

