# Supplementary material for: A founder COL4A4 pathogenic variant resulting in autosomal recessive Alport syndrome accounts for most genetic kidney failure in Romani people
Source: Front Med (Lausanne). 2023 Feb 8;10:1096869. doi: 10.3389/fmed.2023.1096869 (PMC9948603; doi:10.3389/fmed.2023.1096869)
Supplement: Supplementary file 1 [file Data_Sheet_1.docx]

**Supplementary Material 1. List of genes included into the NGS kidney gene panel.**

The original contributions presented in the study are publicly available. This data can be found here: [https://www.ebi.ac.uk/ena/browser//accession number: PRJEB59253].

*ABCA3, ABCC6, ACE, ACTN4, ACVR2B, ACVRL1, ADAMTS13, ADAMTS9, ADD3, AGT, AGTR1, AGTR2, AGXT, AHI1, ALG8, ALMS1, ANKFY1/KIAA1255, ANKS6, ANLN, ANOS1/KAL1, AP2S1, APOL1, AQP2, AR, ARHGAP24, ARHGDIA, ARHGEF18, ARL13B, ARL3, ARL6, ARMC4 , ARMC9, ATP1A2, ATP6AP2, ATP6V0A4, ATP6V1B1, ATP8B1, ATR, AVIL, AVPR2, B9D1/JBTS27/B9/MKS9, B9D2, BAG6, BBIP1, BBS1, BBS10, BBS12, BBS2, BBS4, BBS5, BBS7, BBS9, BICC1, BMP4, BMP7, BMPR1B, BMPR2, BOLA3, BRAF, BSND, C21orf59, C2orf71, C3, C5orf42/CPLANE1, c8orf37, C9orf72, CA2, CASR, CAT, CAV1, CAVIN4, CBLN2, CC2D2A, CCDC103, CCDC114, CCDC141, CCDC151, CCDC28B, CCDC39, CCDC40, CCDC65, CCM2/C7orf22, CCNO, CD2AP, CD46/MCP, CDC5L, CDC73, CELSR2, CENPF, CEP104, CEP120, CEP164, CEP290/NPHP6, CEP41, CEP55, CEP83, CFAP221/PCDP1, CFAP300, CFAP53/CCDC11, CFB, CFC1, CFH, CFHR1, CFHR2, CFHR3, CFHR4, CFHR5, CFI, CFTR, CLCN5, CLCNKA, CLCNKB, CLDN14, CLDN16, CLDN19, CLRN1, CNNM2, COL4A1, COL4A3, COL4A4, COL4A5, COL4A6, COQ2, COQ8B, CRB1, CRB2, CRELD1, CRX, CSF2RA , CSF2RB, CSPP1, CTC1, CTNS, CUL3, CYP11B1, CYP11B2, CYP17A1, CYP24A1, CYP3A5, DACT1, DAW1, DCDC2, DGKE, DMP1, DNAAF1, DNAAF2/KTU, DNAAF3, DNAAF4 /DYX1C1, DNAAF5, DNAH1, DNAH11, DNAH3, DNAH5, DNAH8, DNAH9, DNAI1, DNAI2, DNAJB11, DNAJB13, DNAL1, DRC1, DSTYK, DYNC2H1, DYNC2LI1, DZIP1L, EGF, EHHADH, EIF2AK4, ELMOD2, EMP2, EMX2, ENG, ENPP1, EVC, EVC2, EXOC3L2, EYA1, FAM111A, FAM98C, FGF20, FGF23, FGF8, FGF9, FN1, FOXC1, FOXE1, FOXH1, FOXI1, FOXJ1, FRAS1, FREM1, FREM2, FURIN, FXYD2, G6PC, GALNT3, GANAB, GAPVD1, GAS2L2, GAS8/GAS11/DRC4, GATA3, GATA6, GBE1, GDF1, GDNF, GJA1, GLIS2/NPHP7, GLIS3, GNA11, GNB3, GOSR2, GREB1L/KIAA1772/C18ORF6, GREM1, GRHPR, GRIP1, GUCY2D, HNF1B, HNF4A, HOGA1, HSD11B2, HTRA1, HYLS1, CHD1L, CHRM3, IFIH1, IFT122, IFT140, IFT172, IFT27, IFT43, IFT52, IFT74/CCDC2, IFT80, IFT81, ILK, IMPDH1, INF2/C14ORF173, INPP5E, INTU/KIAA1284, INVS/NPHP2, IQCB1/NPHP5, ITGA8, KANK2/KIAA1518, KAT6B, KCNA1, KCNA5, KCNJ1, KCNJ10, KCNJ13, KCNJ5, KCNK3, KIAA0556/KATNIP, KIAA0586, KIAA0753, KIF14, KIF3B, KIF7, KL, KLHL3, KRIT1, LAMB2, LCA5, LEFTY2, LIN7C, LRAT, LRP4, LRP5, LRRC56, LRRC6, LRRCC1, LZTFL1/BBS17, MAGED2, MAGI2/KIAA0705/AIP1, MAPKBP1, MCIDAS, MEGF8, MEN1, MKKS, MKS1, MMP21, MUC1, MUC5B, MYH9, MYO1E, MYO7A, MYOG, NAT1, NCF1, NEK1, NEK4, NEK8/NPHP9, NFU1, NKX2-1, NKX2-5, NME8, NODAL, NPHP1, NPHP3, NPHP4, NPHS1, NPHS2, NR1H4, NR3C2, NRIP1, NUP107, NUP133, NUP160/NPHS19, NUP205, NUP37, NUP85, NUP93, OCRL, OFD1, PAX2, PAX8, PBX1, PCBD1, PCDH15, PCNT, PCSK5, PDCD10, PDE2A, PDE3A, PDE6D, PHEX, PIBF1, PIH1D3, PKD1, PKD1L1, PKD2, PKHD1, PLCE1, PLK4, PLXND1, PMM2, PNPLA6, POC1A, PRKCD, PRKCSH, PTPN22, PTPRO, RAB23, RD3, RDH12, REN, RET, ROBO2, RPE65, RPGR, RPGRIP1, RPGRIP1L/NPHP8, RSPH1, RSPH3, RSPH4A, RSPH9, SALL1, SALL4, SCARB2, SCN1A, SCNN1A, SCNN1B, SCNN1G, SDCCAG8, SEC61A1, SEC63, SFTPA1, SFTPB, SFTPC, SFTPD, SGPL1, SHROOM3, SIX1, SIX2, SIX5, SLC12A1, SLC12A3, SLC26A4, SLC2A2, SLC34A1, SLC34A3, SLC37A4, SLC3A1, SLC4A1, SLC4A4, SLC5A1, SLC5A2, SLC7A9, SLC9A3R1, SLIT2, SMAD4, SMAD9, SMARCAL1, SOX17, SPAG1, SPEF2, SPRY2, STK36, SUFU, TAC3, TBC1D8B, TBX18, TCTEX1D2, TCTN1/TECT1, TCTN2, TCTN3, TH, THBD, THRA, THRB, TJP2, TMEM107, TMEM127, TMEM138, TMEM216, TMEM231, TMEM237, TMEM256, TMEM67, TOPORS, TRAF3IP1, TRAPPC3, TRIM32, TRPC6, TRPM6, TRPM8, TSC2, TSHR, TTC21B, TTC25, TTC8, TTF1, TULP1, TXNDC15/C5orf14, ULK4, UMOD, UPK3A, USF2, USH1C, USH1G, USH2A, UTRN, VHL, WDPCP/C2ORF86, WDR19, WDR34, WDR35, WDR60, WDR72, WDR73, WHRN, WNK1, WNK4, WNT4, WT1, XDH, XPNPEP3, ZIC3, ZMYND10, ZNF423.*
