# Supplementary material for: A founder COL4A4 pathogenic variant resulting in autosomal recessive Alport syndrome accounts for most genetic kidney failure in Romani people
Source: Front Med (Lausanne). 2023 Feb 8;10:1096869. doi: 10.3389/fmed.2023.1096869 (PMC9948603; doi:10.3389/fmed.2023.1096869)
Supplement: Supplementary file 2 [file Data_Sheet_2.docx]

**Supplementary Material S2. Benign variants in patients with homozygous p.Gly533Arg in *COL4A4***

**Variants in *COL4A3* (Ref Seq NM_000091.4):**

c.45-99C>T (rs7579991)

c.280-39_40insG

c.324+73C>T (rs6750210)

c.422T>C, p.Leu141Pro (rs10178458)

c.441+146G>T (rs10168566)

c.441+150G>T (rs10168567)

c.442-88A>G (rs4321358)

c.468+139C>T (rs12612699)

c.485A>G, p.Glu162Gly (rs6436669)

c.765+49T>G (rs12621551)

c.987+35T>G (rs73993878)

c.1576-60G>A (rs6436672)

c.1195C>T, p.Leu399= (rs10205042)

c.2223+100G>T (rs6729152)

c.2374+82A>G

c.2881+46A>G (rs6436672)

**Variants in *COL4A4* (Ref Seq NM_000092.4):**

c.490-121T>G (rs12465531)

c.595-111T>C (rs6436654)

c.657+48A>G (rs73082223)

c.657+62G>A (rs59938187)

c.657+67A>G (rs58363082)

c.658-39T>C (rs12475686)

c.871-37_60del23

c.1444C>T, p.Pro482Ser (rs2229814)

c.2545+46A>T (rs7567796)

c.2545+51A>G (rs7567789)

c.2545+140T>C (rs4566357)

c.2861-142A>G (rs3769643)

c.2861-112G>C (rs3769644)

c.3011C>T, p.Pro1004Leu (rs1800517)

c.3151-22T>C

c.3214+129G>C (rs2272203)

c.3214+159A>G (rs2272202)

c.3215-66C>T (rs2272200)

c.3594G>A, p.Gly1198= (rs10203363)

c.3684G>A, p.Lys1228= (rs2229812)

c.3817+9G>C (rs13423714)

c.3973+34T>C (rs1917127)

c.4080G>A, p.[Pro1360=] (rs2228556)

c.4091-36G>A (rs3752896)

c.4217-24C>T (rs3752896)

c.4522+72G>A (rs4423583)

c.4523-8T>C (rs13419076)

c.4548A>G, p.Val1516= (rs2228555)

c.4932C>T, p.Phe1644= (rs2228557)
